# Internet Interventions or Patient Education Web Sites? – Author’s Reply

**DOI:** 10.2196/jmir.8.3.e19

**Published:** 2006-09-29

**Authors:** Cicely Kerr, Elizabeth Murray, Fiona Stevenson, Charles Gore, Irwin Nazareth

**Affiliations:** ^2^The Hepatitis C TrustLondonUnited Kingdom; ^1^Department of Primary Care and Population SciencesLondonUnited Kingdom

We agree with Ritterband and Thorndike that the terminology and definitions in this field are confusing, and should be clarified and standardized. We would be happy to work with them and others on such a project.

However, they are mistaken in their belief that the interventions used in our study were mostly patient education websites [1]. The interventions were carefully selected to fulfil the criteria for Interactive Health Communication Applications (IHCAs); namely that they were computer-based programs that combined health information with at least one interactive component, such as decision support, behavior change support or peer support. For example, heartcenteronline.com contains interactive self-assessment tools, as a support for behavior change, as well as online peer support, in the form of both personal stories and online chat groups. Similarly, alzheimersdisease.com contains interactive tools and online peer support, in the form of an e-mail "buddy" arrangement.

This definition of an IHCA was provided in the paper, as were the criteria for choice of IHCA used in this study. As IHCA is a somewhat clumsy term, we preferred to use the more intuitive term “Internet interventions” to refer to web-based IHCAs.

The other major point raised by Ritterband and Thorndike refers to our chosen methodology. It is the nature of qualitative research to work in-depth with small samples. We believe the combination of a qualitative research design allowing participating patients and caregivers to generate and define criteria, followed by a further validation exercise allowing them to check we have identified important criteria, is a particular methodological strength [2-3]. We did not set out to produce a list of generic criteria and in our analysis we were alert to the likelihood that patients and caregivers managing different chronic conditions would have different quality criteria. Instead, however, it was striking how similar needs and quality criteria were across groups.

This is the basis for the generic quality criteria described in the paper and we question Ritterband and Thorndike’s assertion that an intervention for people with diabetes would self-evidently be completely different to one for insomnia. Our work concentrates on people with long-term conditions. Lorig has proposed that people with long-term conditions face three tasks (medical management, emotional management and role management) irrespective of the type of condition. These tasks require specific skills, such as problem solving, decision-making, finding and utilizing resources, forming partnerships with health professionals and taking action. Lorig postulates that enhancing self-efficacy, (i.e. a person’s belief in their capacity to carry out a specific action) is key to enhancing self-care skills [4]. Based on this theory, interventions designed to enhance self-care skills in people with long-term conditions should target these skills and aim to enhance self-efficacy. The specific content of an intervention will differ according to the condition targeted, but the theoretical basis, and hence the core components (e.g. tailored information, decision-support, action planning, emotional support) may well be similar.
